# Comparative evaluation of rapidity of action of benzydamine hydrochloride 0.3% oromucosal spray and benzydamine hydrochloride 3 mg lozenges in patients with acute sore throat: A phase IV randomized trial

**DOI:** 10.1097/MD.0000000000033367

**Published:** 2023-03-31

**Authors:** Carmelina Valerio, Giorgio Di Loreto, Enrica Salvatori, Agnese Cattaneo

**Affiliations:** a Angelini Pharma S.p.A – Global Medical Department, Rome, Italy.

**Keywords:** analgesia, benzydamine hydrochloride, inflammation, pain, pharyngitis, sore throat

## Abstract

Acute sore throat (ST) can occur as part of a common cold of viral origin or caused by pharyngeal bacterial pathogens. The majority of patients with acute ST complain of pain on swallowing and dry scratchiness which can have a negative impact on the quality of life (QoL). This study aimed to evaluate the time to pain relief in patients with acute ST, following a single administration of benzydamine hydrochloride (HCl) 0.3% oromucosal spray or benzydamine HCl 3 mg lozenges.

**Methods:**

This multicenter, randomized, active-controlled, open label, parallel-group, international phase IV study was conducted at 12 investigational centers in Poland, Hungary, and Russian Federation. The study population consisted of 363 adult patients with recent onset (≤3 days) of ST and a diagnosis of tonsillopharyngitis. The primary endpoint was to assess the efficacy of benzydamine HCl in ST pain relief at 2 minutes after a single-dose administration. Secondary endpoints included, among others, the assessment of a first perceived ST relief at 1 minute after a single-dose administration of benzydamine HCl spray or lozenge.

**Results:**

Both the spray and lozenges are effective in providing a ST relief starting already at 2 minutes after a single administration, with an effect lasting up to up to 4 hours. Clinical efficacy after 7 days of treatment and a good safety profile were also demonstrated.

**Conclusion:**

Anesthetic and analgesic properties of benzydamine spray and lozenges effectively addressed the patient priority of a rapid relief of symptoms of upper respiratory tract infections (URTI).

## 1. Introduction

Acute sore throat (ST) often occurs as part of a common cold of viral origin or caused by pharyngeal bacterial pathogens.^[1,2]^ Noninfectious physicochemical factors may also be a trigger of painful pharyngitis.^[3]^ The majority of patients with acute ST complain of pain on swallowing, dry scratchiness, cough and flu-like symptoms that can have a negative impact on the quality of life (QoL).^[4]^ ST is often caused by an inflammation of the pharynx, tonsils or nasopharynx related to the release of inflammatory mediators, including bradykinin and prostaglandins, following the local responses to cell damage, and that exert their effect on sensory nerves in the airways.^[5]^ In addition, pro-inflammatory cytokines, such as interferon-α, may induce behavioral changes and be associated with sickness symptoms.^[6]^

Symptomatic relief of pain is the main objective of most over-the-counter treatments available for the self-management of ST, with different formulations and characteristics.^[7]^ In particular, medicated throat lozenges have the advantage over sprays and gargles of being slow-releasing, ensuring continuous delivery of the active ingredients to all affected areas of the throat and over a prolonged period of time,^[8]^ while oral sprays are more effective in coating the oropharynx than oral rinses.^[9]^

Benzydamine hydrochloride (HCl) is an indolic nonsteroidal antiinflammatory drug endowed with some peculiar properties not shared by other NSAIDs, such as local anesthetic and analgesic activities, together with antifungal and antimicrobial properties.^[10]^ Benzydamine HCl is a weak inhibitor of the synthesis of prostaglandins and a strong inhibitor of proinflammatory cytokines, particularly tumor necrosis factor-α, interleukin-1β and monocyte chemoattractant protein-1.^[11]^ Of interest, benzydamine HCl can exert some of its antiinflammatory activities by reducing the vascular changes occurring during inflammation, as well as through the inhibition of neutrophil degranulation with a membrane-stabilizing activity.^[10]^ Locally applied benzydamine HCl also exhibits marked local anesthetic properties, with a rapid effect on pain related to the interaction with cationic channels.^[12]^ Its topical application produces sufficient drug concentrations in the inflamed area^[13]^ with low systemic absorption, thereby limiting unrequired systemic exposure to the drug.^[14]^ In addition, benzydamine HCl revealed the ability to block the neuronal excitability,^[15–17]^ thus supporting the synergistic and complementary effects of benzydamine HCl in the treatment of local inflammatory symptoms as well as in the painful processes, through a reduction of the inflammation cascade and of the inflammatory-mediated neuronal signaling.

Topical formulations of benzydamine HCl are available for the treatment of several oropharyngeal conditions,^[14]^ proving to be safe and efficient, not only in the symptomatic treatment of pain and irritative/inflammatory conditions of the oropharynx, but also in various conditions ranging from posttonsillectomy pharyngitis or radiomucositis, to throat irritation and/or dysphagia induced by intubation.^[18,19]^

Acute pharyngitis and tonsillitis are not life-threatening conditions but may have profound negative influence on QoL.^[20]^ Patients with ST often complain of odynophagia, throat swelling, and dysphagia,^[20]^ and may also report a broad range of other functional painful qualities of ST.^[21]^ From the patient perspective, a rapid relief of symptoms is the most important aspect of treating upper respiratory tract infections (URTI). In this setting, we performed a phase IV study (NCT04941976; EudraCT no.: 2019-003257-29) aimed at assessing the speed of pain relief in patients with acute ST, following the administration of benzydamine HCl 0.3% oromucosal spray or benzydamine HCl 3 mg lozenges. Safety and efficacy up to a 7-days treatment period were also evaluated. Finally, any differences between the 2 formulations, in terms of pain relief, duration of the analgesic effect, and effect on other symptoms related to ST were described as well.

## 2. Methods

### 2.1. Study design

A multicenter, randomized, active-controlled, open label, parallel-group, international study was conducted from August 2020 to June 2021 in 12 investigational sites from different sources (general practices, hospitals, and outpatients’ facilities) located in Hungary, Poland and Russian Federation to evaluate the efficacy and the safety of benzydamine HCl 0.3% oromucosal spray and benzydamine HCl 3 mg lozenges in patients with acute ST.

Adult patients (18–75 years) of both sexes, with recent onset of ST (≤3 days), at least 1 symptom of URTI in the previous 24 hours on the URTI questionnaire, a ST pain intensity score ≥ 60 mm on Sore Throat Pain Intensity Scale (STPIS) and an objective diagnosis of tonsillopharyngitis confirmed by a score ≥ 5 on the Tonsillo-Pharyngitis Assessment (TPA) were enrolled in the study. Three visits were scheduled during the study (V0, V1, and V2), and activities at each visit were performed as scheduled in Table 1. The trial was approved by Institutional Review Board/Ethics Committees and was conducted in compliance with study protocol, Good Clinical Practices, Declaration of Helsinki and applicable regulatory requirements. All patients signed the Informed consent form and the Declaration of consent for processing of personal data.

**Table 1:** Schedule of BePaiR study activities.

At investigational site DAY 0										At home from D 0 to D 6	At investigational site D 7 (+2)
Visit	V0 screening/baseline	V1 efficacy evaluation									V2/ETTV final visit
Time	0	1 min	2 min	5 min	10 min	15 min	30 min	60 min	120 min		
Informed consent and personal data processing	X										
Demographic data and medical history	X										
Physical examination and vital signs	X										X
Previous treatments	X										
Concomitant treatments	X									X	X
Pregnancy test	X										
Rapid strep test	X										
Inclusion/Exclusion criteria	X										
URTI questionnaire	X										
TPA	X										X
STPIS	X									X	X
DSS	X			X	X	X	X	X	X		
SwoTS	X			X	X	X	X	X	X		
QuaSTI	X							X	X		
Randomization	X										
Drug administration	X									X	
Adverse events	X	X	X	X	X	X	X	X	X	X	X
STRRS		X	X	X	X	X	X	X	X	X	
Drug delivery									X		
Patient card delivery									X		
Patient diary delivery									X		
Patient diary collection and IMP return											X
PSQ											X

DSS = difficulty swallowing scale, IMP = investigational medicinal product, PSQ = patient satisfaction questionnaire, QuaSTI = qualities of sore throat index, STPIS = sore throat pain intensity scale, STRRS = sore throat relief rating scale, SwoTS = swollen throat scale, TPA = Tonsillo-Pharyngitis assessment, URTI = upper respiratory tract infection.

### 2.2. Treatment administration

This was the first study that directly compared the relief provided by benzydamine HCl, using 2 different formats in the same study population. Patients were randomized to treatment according to the order of recruitment either to 1 dose of benzydamine HCl spray oromucosal solution (test product - 4 nebulizations corresponding to 2.04 mg of benzydamine HCl), or to one 3 mg lozenge of benzydamine HCl (reference therapy - corresponding to 2.68 mg of benzydamine HCl), both administered at the investigational site. The efficacy evaluations started immediately after the nebulization or after the complete lozenge dissolution (taking an average time of 9.12 minutes) (Table 1). Subsequently, the patients took the test or the reference products at home, according to the local summary of product characteristics, for a maximum of 7 days from the first application and up to the symptoms’ resolution.

### 2.3. Study objectives

The primary objective of the study was to assess the efficacy of benzydamine HCl (spray or lozenges) in ST pain relief at 2 minutes (T2min) after a single dose administration.

The secondary objectives included the evaluation, after a single-dose administration of benzydamine HCl, of a first perceived ST relief at 1 minute; the meaningful ST relief and the effect on difficult swallowing and sensation of swollen throat at T5min; T10min; T15min; T30min; T60min; T120min; the effect on different characteristics of ST pain and discomfort after 60 minutes and 120 minutes; the ST intensity across the study, the pharyngeal inflammation status and the patient satisfaction at the end of the treatment; safety assessment.

### 2.4. Study endpoints

The primary endpoint of the study was the percentage of responders defined as patients reporting at least a “slight relief” (Sore Throat Relief Rating Scale [STRRS] score ≥ 1) at T2min after the first application of benzydamine HCl (spray or lozenges).

As secondary endpoints, the comparison between the 2 treatment groups was assessed to determine: the percentage of patients recording a first perceived ST relief (STRRS score ≥1) assessed at T1min; the percentage of patients recording a meaningful ST relief (STRRS score ≥ 3) assessed from T5min to T120minutes after a single dose administration (Table 1); the STRRS change from 1 minute up to 4 hours postdose. Other secondary endpoints were the assessment of changes from baseline in the Difficulty Swallowing Scale (DSS), the Swollen Throat Scale (SwoTS), the Qualities of Sore Throat Index (QuaSTI), the ST pain intensity through the STPIS assessment, the evaluation of clinical effects at the end of the treatment period through a TPA and the patient global treatment evaluation at the end of the treatment period through a Patient Satisfaction Questionnaire (PSQ). The safety assessment was also performed. Tests and questionnaires used for the evaluation of study endpoints are described in detail in Table S1, Supplemental Digital Content 1, http://links.lww.com/MD/I709 and were performed as scheduled in Table 1.

### 2.5. Patient populations

Efficacy was evaluated in both the modified intent-to-treat (m-ITT) population, including all randomized patients who took at least 1 dose of the investigational medicinal product (IMP) and performed the T2min efficacy assessment with STRRS, and the per-protocol (PP) population, including all randomized patients who took at least the first dose of the IMP and performed all Day 0 (up to 120 minutes) STRRS evaluations, with no major protocol violations. As this was a noninferiority study, the PP population was used as the primary analysis population. In order to judge the validity of the PP results, the noninferiority analysis was repeated in the m-ITT population. The safety population (SP), comprising all those who took at least 1 dose of the study medication, was used to evaluate safety data and demographic, baseline, and treatment characteristics. All adverse events (AEs) and treatment emergent AEs (TEAEs) were coded according to MedDRA dictionary (version 23.1). For exploratory purposes only, an additional modified-PP (m-PP) population was included to describe secondary efficacy endpoints after 120 minutes. The m-PP population was defined by the PP population with the exclusion of patients who took concomitant treatments or additional doses of IMP during the at-home period or 3 lozenges before V2 on Day 7.

### 2.6. Statistics

Benzydamine HCl spray was considered not inferior to benzydamine HCl lozenges if the lower limit of the 2-sided 95% confidence interval (95% CI) of the difference in responder rates between the benzydamine HCl formulations, defined as % of patients reporting at least a “slight relief” (STRRS score ≥ 1) at T2min, did not exceed the threshold of −10%. Assuming a responder rate, defined as percentage of patients reported at least a slight relief (STRRS score ≥ 1), equal to 90% in the benzydamine HCl lozenges arm at T2min, a sample size of 178 patients per group (with a drop-out rate of 20%) was required to claim the noninferiority between treatment groups, using a 2-tailed confidence level of 95%. In this way, the lower bound of the confidence interval (CI) of the difference in the responder rates between the treatment groups should not exceed the threshold of −10.0% with a power of 80%.

The Cochran-Mantel-Haenszel test was applied on percentage of patients with a STRRS score ≥ 1 at 1 minute after the single dose administration and on percentage of patients with a STRRS score ≥ 3 at 5, 10, 15, 30, 60, and 120 minutes after the single dose administration.

ANCOVA or ANOVA were applied on: the sum of pain intensity differences (SPID) for DSS and SwoTS, estimated as the area under the pain intensity difference (PID) versus time curve up to 2 hours postdose (the PID was calculated by subtracting the baseline pain intensity score from the actual pain intensity score); the total pain relief for STRRS, estimated as the area under the pain relief versus time curve by adding the pain relief scores observed 2 to 240 minutes postdose; QuaSTI at 60 and 120 minutes postdose; TPA; STPIS. Covariate used in ANCOVA was the baseline value. Scores were reported as mean ± standard deviation (SD).

Descriptive statistics was applied to PSQ at the end of the treatment, to vital signs and their change from V0 and to change from baseline in physical examination presented by treatment group.

Confidence intervals and statistical tests for secondary endpoints were of descriptive nature.

## 3. Results

This phase IV study aimed to generate new clinical data about the speed of relief provided by a single application of Tantum Verde oromucosal spray (benzydamine HCl 0.3%) or Tantum Verde lozenges (benzydamine HCl 3 mg).

**Figure 1.**
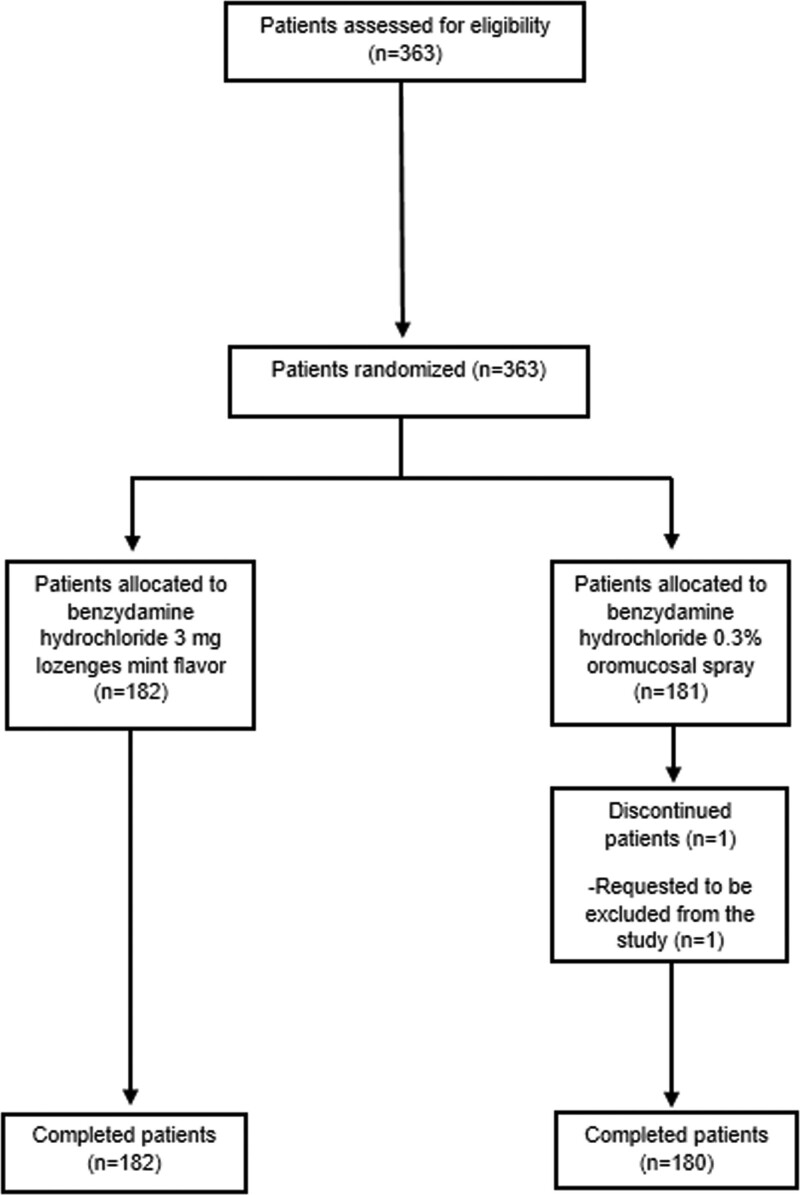
Patients disposition in BePaiR study.

A total of 363 adult patients with recent onset of ST (≤3 days) and a confirmed diagnosis of tonsillopharyngitis were randomized to benzydamine HCl 0.3% spray, (n = 181) or benzydamine HCl 3mg lozenges (n = 182) (Fig. 1) and took at least 1 dose of the IMP (SP) (refer to Table S2, Supplemental Digital Content 2, http://links.lww.com/MD/I710 for size of the 4 populations used for data analysis).

### 3.1. Primary endpoint of the study

A total of 355 (174 benzydamine HCl 0.3% spray and 181 benzydamine HCl 3 mg lozenges) patients entered in the efficacy analysis as PP population and 363 patients as m-ITT population (181 benzydamine HCl 0.3% spray and 182 benzydamine HCl 3 mg lozenges). Based on the results, benzydamine HCl spray resulted not inferior to benzydamine HCl lozenges, as the difference between the percentage of patients reporting at least a “slight relief” (STRRS score ≥ 1) at 2 minutes after the administration of benzydamine HCl spray or benzydamine HCl lozenges, did not exceed the threshold of −10% in both the PP (95% CI, −5.18; 6.73) and m-ITT (95% CI, −4.78; 6.88) populations (Table 2). Consequently, the primary efficacy endpoint of the study was met. In addition, a high response rate was observed (91.4% responders in the benzydamine HCl spray arm and 90.6% responders in the benzydamine HCl lozenges arm in the PP population and 91.7% responders in the benzydamine HCl spray arm and 90.7% responders in the benzydamine HCl lozenges arm in the m-ITT population), confirming the effectiveness of benzydamine HCl in providing a rapid pain relief.

**Table 2:** Efficacy analysis of Benzydamine HCl 0.3% spray and Benzydamine HCl 3 mg lozenges in PP and m-ITT populations.

	BNZ spray (n = 174)	BNZ lozenges (n = 181)	BNZ spray (n = 181)	BNZ lozenges (n = 182)
	PP population		m-ITT population	
Responders	159 (91.4%)	164 (90.6%)	166 (91.7%)	165 (90.7%)
Non responders	15 (8.6%)	17 (9.4%)	15 (8.3%)	17 (9.3%)
95% CI	−5.18; 6.73		−4.78; 6.88	

BNZ = benzydamine, HCl = hydrochloride, m-ITT = modified intent-to-treat, PP = per-protocol.

### 3.2. Secondary endpoints of the study

A first pain relief (STRRS score ≥ 1) was perceived already at 1 minute after the administration of the benzydamine HCl spray and lozenge: high responder rates were recorded for both the treatment groups (m-ITT population: 77.9% for spray/87.4% for lozenges; PP population: 77.6% for spray/87.8% for lozenges), with significantly more responders in benzydamine HCl lozenges arm in comparison with benzydamine HCl spray arm (*P* = .0041 in m-ITT population and *P* = .0019 in PP population).

**Figure 2.**
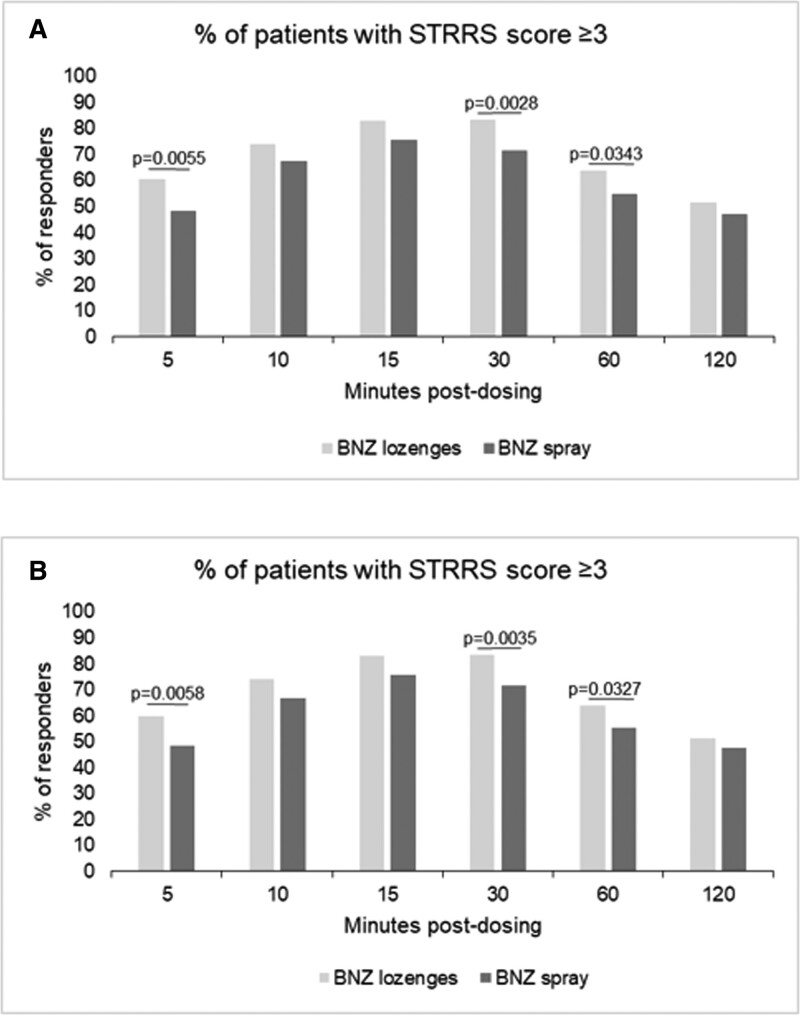
Percentage of patients with a STRRS score ≥ 3 at different time points after a single dose administration of benzydamine HCl spray or benzydamine HCl lozenges as secondary endpoint of BePaiR study. A significant difference in the percentage of responders between the treatment with benzydamine HCl spray and benzydamine HCl lozenges was detected at 5, 30, and 60 min post dosing. A: PP population; B: m-ITT population. HCl = hydrochloride, m-ITT = modified intent-to-treat, STRRS = sore throat relief rating scale.

The prompt relief provided by the application of benzydamine HCl was also confirmed by the high responder percentage of patients reporting a meaningful ST relief (STRRS score ≥ 3). The percentage of patients who experienced a meaningful pain relief was considerable already at T5min after the single dose application and increased at T10min, with a peak at T15min and T30min and a decrease from T60min. However, at T120min, about half of the participants still showed significant pain relief in both the treatment arms. In the benzydamine HCl lozenges arm, the percentage of patients experiencing the ST relief was significantly higher at 5, 30 and 60 minutes post single-administration in comparison to the benzydamine HCl spray arm (Fig. 2). Comparable results were obtained in PP and m-ITT populations (Fig. 2). The mean total pain relief for STRRS from 2 to 240 minutes postdose did not show significant differences between benzydamine HCl spray and benzydamine HCl lozenges in m-ITT, PP and m-PP populations (Figure S1, Supplemental Digital Content 3, http://links.lww.com/MD/I711).

**Figure 3.**
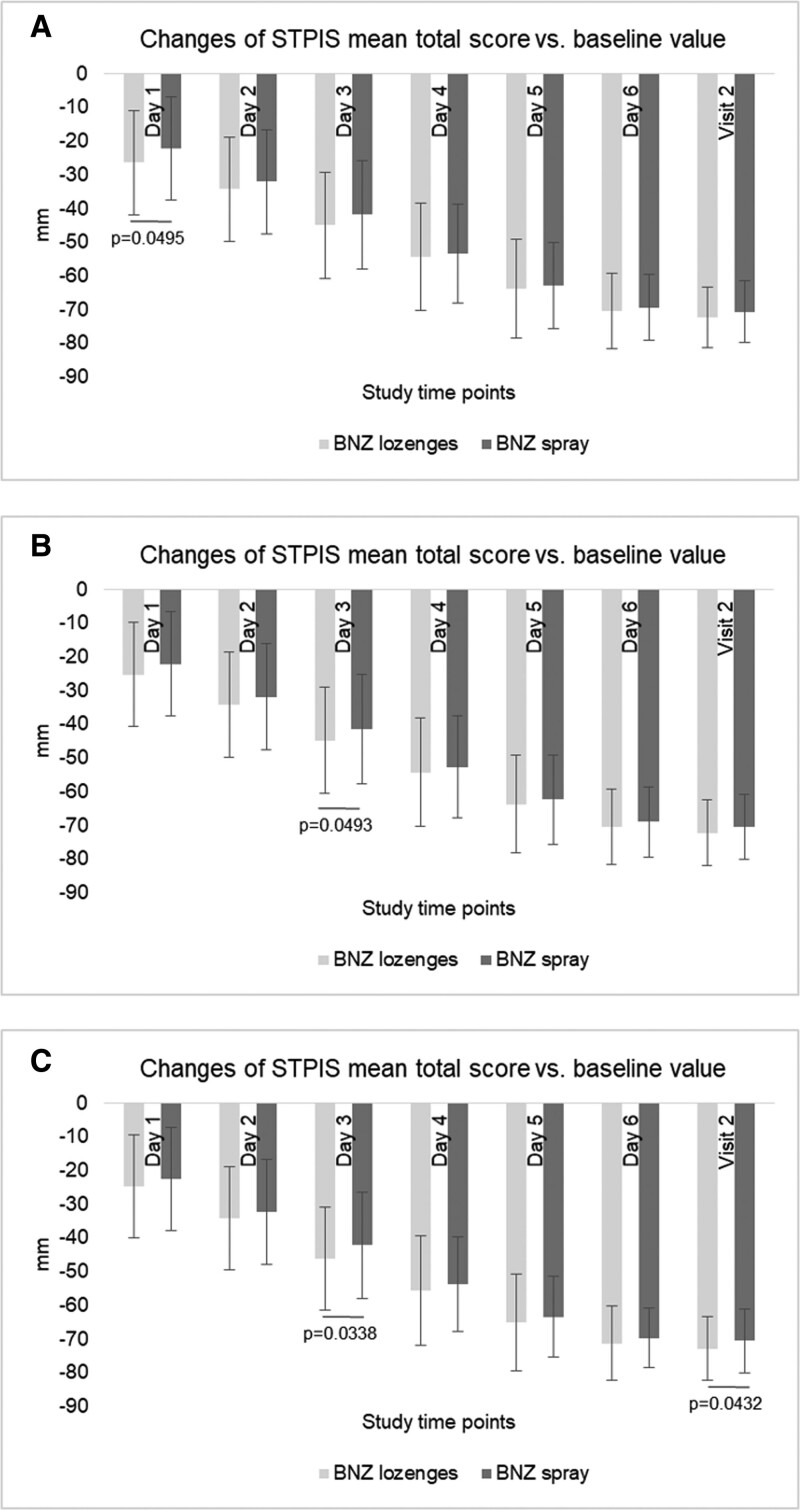
Changes of STPIS total score (mean ± SD) versus baseline value at different time points of BePaiR study after administration of benzydamine HCl spray or benzydamine HCl lozenges. All changes of STPIS scores at different time points were statistically significant *vs* the baseline value (*P* < .0001). In addition, significant changes between the benzydamine HCl spray and the benzydamine HCl lozenges arms were detected at D 1 in PP population, at Day 3 in m-ITT and m-PP populations and at Visit 2 in m-PP population, as indicated. A: PP population; B: m-ITT population; C: m-PP population. HCl = hydrochloride, m-ITT = modified intent-to-treat, m-PP = modified per-protocol, PP = per-protocol, STPIS = sore throat pain intensity scale.

At the end of the week of treatment, in the m-ITT population, the total improvement in STPIS average scores was by 70.41 mm ± 9.59 mm and 72.19 mm ± 9.86 mm in benzydamine HCl spray arm and in benzydamine HCl lozenges arm, respectively, also confirmed in the PP and the m-PP population analysis. A steady and statistically significant decrease in ST pain intensity until V2 was observed every day in both benzydamine HCl spray and benzydamine HCl lozenges arms and for PP, m-ITT and m-PP populations (*P* < .0001 vs V0), showing that both treatments provided a significant reduction of ST up to 1 week of treatment (Fig. 3). Statistically significantly changes in STPIS scores resulted lower in favor of benzydamine HCl lozenges at different time points in the 3 populations (Fig. 3).

The TPA assessment in m-ITT population documented an improvement of the score from V0 to V2 by 8.38 ± 2.40 for benzydamine HCl spray arm and 8.47 ± 2.43 for benzydamine HCl lozenges arm (V2 vs V0, *P* < .0001), with no statistically significant difference between the 2 arms. This improvement was confirmed in PP and m-PP population analysis.

The SPID analysis of data from the SwoTS (0–120 minutes postdosing) revealed an improvement of the sensation of swollen throat of 46.15 mm ± 39.21 mm for benzydamine HCl spray and 52.27 mm ± 38.93 mm for benzydamine HCl lozenges in the m-ITT population and an improvement of 46.48 mm ± 39.88 mm for benzydamine HCl spray and 52.11 mm ± 38.98 mm for benzydamine HCl lozenges in the PP population *vs* the baseline values. The SPID analysis of data from the DSS (0–120 minutes postdosing) revealed an improvement of the difficulty in swallowing of 49.92 mm ± 41.41 for benzydamine HCl spay and 50.18 mm ± 41.69 mm for benzydamine HCl lozenges in the m-ITT population and an improvement of 46.48 mm ± 39.88 mm for benzydamine HCl spray and 52.11 mm ± 38.98 mm for benzydamine HCl lozenges in the PP population *versus* the baseline values. The efficacy of the 2 treatments can be considered comparable as no statistically significant difference was found between the 2 treatment arms.

For all items of the QuaSTI (listed in Table S1, Supplemental Digital Content 1, http://links.lww.com/MD/I709), both benzydamine HCl treatments showed a similar and statistically significant effectiveness in reducing symptomatology at T60min and T120min relative to baseline in m-ITT and PP populations (*P* < .0001). Results also showed the absence of significant differences between the 2 treatment arms, with the exception of QuaSTI agonizing score change at T60min *versus* V0, showing a significant difference in favor of benzydamine HCl lozenges in m-ITT and PP populations (*P* = .0199).

Moreover, TPA total score demonstrated that all features of pharyngeal inflammation had a statistically (*P* < .0001) and clinically improvement in both treatment arms in m-TT, PP and m-PP population and in comparison to baseline assessments, confirming the efficacy of benzydamine HCl in reducing ST pain and inflammation.

Finally, results of the PSQ revealed that more than 90% of participants in m-ITT, PP and m-PP populations expressed a positive opinion regarding the study treatment. The majority of patients agreed/strongly agreed that the IMPs led to a rapid improvement of ST symptoms, both formulations were easy to use and suitable for daily use meeting the patient expectations.

### 3.3. Safety evaluation

Safety analyses were based on the SP and was evaluated by monitoring frequency of AEs and changes from baseline in physical examination and vital signs. Both benzydamine HCl formulations were well tolerated and showed a good safety profile, without clinically significant abnormalities. No clinically relevant changes from baseline or relevant differences between treatment groups were observed for vital signs and physical examination.

Moreover, 30 TEAEs were documented in 22 patients (one in benzydamine HCl spray arm, 21 in benzydamine HCl lozenges arm); 9 TEAEs were classified as mild, and all were resolved. None of them was serious or led to withdrawal.

## 4. Discussion

Acute pharyngitis and tonsillitis are not life-threatening conditions but may have a profound negative effect on QoL.^[22]^ Adults have in average 2 to 4 URTI/year, usually occurring during the colder months, rising to 6 to 8 infections/year in children because of their immunological naivety,^[1]^ and are a common reason for primary care consultations.^[23]^ The therapeutic options available for the treatment of ST are aimed not only to eradicate the causal agents and produce a clinical cure, but also to increase patients’ QoL.^[24]^ In particular, local therapies are useful for symptomatic treatment of ST as they allow direct application to the painful area, providing rapid and effective pain relief, with a reduced risk of toxicity when compared with systemic treatments.^[25]^ The local anesthetic activity of benzydamine HCl has been shown to be extremely useful in the treatment of painful throat and mouth conditions, mainly due to rapid pain relief provided.^[26]^ The study results confirm that initial rapid relief from ST pain is perceived by most patients as early as 2 minutes after administration when benzydamine HCl is delivered as either a lozenge or a spray, addressing the patients’ priority of a rapid relief of symptoms of URTI. Evaluation of secondary endpoints showed nearly similar findings for both benzydamine HCl treatment arms. A significant difference in favor of the benzydamine HCl lozenges versus the spray was observed for patients who experienced the first perceived pain relief after 1 minute. This result could be explained considering that efficacy evaluations for lozenges started only when complete dissolution occurs. Consequently, by the time of the first assessment patients had already perceived an initial relief. Conversely, the pain relief assessment carried out for the spray was performed immediately after its administration. A clinical and statistically significant reduction from baseline in pain intensity, pharyngeal inflammation and other pain dimensions was achieved by both formulations every day and also over a period of 1 week.

Of note, the appropriate use, and the effectiveness of benzydamine HCl in the treatment of ST has been recently reinforced by a Delphi-based international consensus involving pharmacists and general practitioners.^[27]^ In addition, a cross-national survey among pharmacists and general practitioners revealed that benzydamine HCl is largely recognized and recommended as a suitable topical treatment of ST and other inflammatory conditions of the oral cavity.^[28]^

## 5. Conclusion

Benzydamine HCl as oromucosal spray 0.3% and 3 mg lozenges confirmed its value providing a rapid relief from symptoms of ST with a high safety profile and product acceptability, thus representing a valuable tool in the clinical practice for the treatment of ST, allowing the patients to choose between 2 equivalent benzydamine HCl formulations according to their own preferences.

## Acknowledgments

Medical writing support was provided by Amalia Forte, PhD, on behalf of Menthalia, Naples, Italy.

## Author contributions

**Conceptualization:** Carmelina Valerio, Enrica Salvatori.

**Data curation:** Giorgio Di Loreto.

**Formal analysis:** Giorgio Di Loreto.

**Methodology:** Carmelina Valerio, Giorgio Di Loreto.

**Project administration:** Carmelina Valerio.

**Software:** Giorgio Di Loreto.

**Supervision:** Enrica Salvatori, Agnese Cattaneo.

**Visualization:** Carmelina Valerio.

**Writing – review & editing:** Carmelina Valerio, Giorgio Di Loreto, Enrica Salvatori.

## Supplementary Material






