# Supplementary material for: Comparative evaluation of rapidity of action of benzydamine hydrochloride 0.3% oromucosal spray and benzydamine hydrochloride 3 mg lozenges in patients with acute sore throat: A phase IV randomized trial
Source: Medicine (Baltimore). 2023 Mar 31;102(13):e33367. doi: 10.1097/MD.0000000000033367 (PMC10063282; doi:10.1097/MD.0000000000033367)
Supplement: Supplementary file 1 [file medi-102-e33367-s001.pdf]

Supplemental Table 1: Tests and questionnaires undertaken for the evaluation of primary and secondary endpoints of BePaiR Study

Acronym	Definition	Description
**DSS**	**Difficulty Swallowing Scale**	The DSS is a visual analogic scale that assesses the difficulty of swallowing.<br>It consists of a continuous horizontal line of 10 cm in length where 0= “not difficult” and 100= “very difficult”.<br>It was self-administered by the patient at V0 (before drug intake – T0) and at V1: T5min, T10min, T15min, T30min, T60min and T120 minutes.<br>The patient was instructed to swallow and, as soon as swallowed, to assess the Difficulty Swallowing.
**PSQ**	**Patient Satisfaction Questionnaire**	The PSQ consists of 11 items to assess the patient global judgment on the study treatment. The patient was asked to give a feedback about the drug performance, its organoleptic properties, its -friendly use, etc.<br>The patient expressed his/her opinion choosing from the following items: “Strongly disagree”, “Disagree”, “Agree”, “Strongly agree”.<br>The PSQ was completed by the patient at V2.
**QuaSTI**	**Qualities of Sore Throat Index**	The QuaSTI consists of a 10-item index of qualities of sore throat: agonizing, burning, difficulty swallowing, dry, husky/hoarse voice, irritated/scratchy, like a lump in the throat, raw, swollen, tight which are rated on an ordinal scale of 0–10, where 0= “not at all” and 10= “a lot”.<br>It was completed by the patient at V0 (baseline) and at V1 (T60min and T120min).
**STPIS**	**Sore Throat Pain Intensity Scale**	The STPIS is a visual analogic scale (VAS) that assesses the intensity of ST. It consists of a continuous horizontal line of 10 cm in length, with ends labelled as the extremes of pain, from 0 (left side): “no pain”, to 10 (right side): “pain as bad as it could be”. The patient was asked to place a line with a pen perpendicular to the VAS line and intersecting the VAS line at the point that represents the current pain intensity. Using a ruler, the score was determined by measuring the distance (mm) on the 10-cm line between the “no pain” anchor and the patient’s mark, starting the measurement from the “no pain” anchor, providing a range of scores from 0 to 100 mm. A higher score indicates greater pain intensity.<br>It was self-administered by the patient at V0 (before drug intake), at home (each 24 hours after the first study medication intake, until the symptoms resolution or up to Day 6) and at V2, or ETTV (if applicable). The patient was instructed to swallow and then to complete the rating scale as soon as swallowed, at each time point.
**STRRS**	**Sore Throat Relief Rating Scale**	The STRRS is a 7-point categorical scale that assesses the local analgesic effect.<br>It consists of the following 7 items: 0= “no relief”, 1= “slight relief”, 2= “mild relief”, 3= “moderate relief”, 4= “considerable relief”, 5= “almost complete relief”, and 6= “complete relief”.<br>The scale was self-administered by the patient at each time points of V1: T1min, T2min, T5min, T10min, T15min, T30min, T60min T120min and at home at T240min from the first drug administration. The patient was instructed to swallow and then to complete the rating scale as soon as swallowed, at each time-point.
**SwoTS**	**Swollen Throat Scale**	The SwoTS is a 100 mm visual analogical scale that assesses the swollen feeling by the patient.<br>It consists of a continuous horizontal line of 10 cm in length where 0= “not swollen” and 100= “very swollen”.<br>It was self-administered by the patient at V0 (before drug intake) and at V1: T5min, T10min, T15min, T30min, T60min and T120 min.<br>The patient was instructed to swallow and, as soon as swallowed, indicate how swollen his/her throat felt.
**TPA**	**Tonsillopharyngitis Assessment**	The TPA is an index of seven objective features of pharyngeal inflammation: oral temperature, oropharyngeal colour, size of tonsils, number of oropharyngeal enanthems (vesicles, petechiae or exudates), largest size of anterior cervical lymph nodes, number of anterior cervical lymph nodes, and maximum tenderness of some anterior cervical lymph nodes. A maximum possible score of 21 can be recorded.<br>It was assessed by the investigator at V0 (baseline) and at V1 (T60min and T120min).
