# Supplementary material for: Comparative evaluation of rapidity of action of benzydamine hydrochloride 0.3% oromucosal spray and benzydamine hydrochloride 3 mg lozenges in patients with acute sore throat: A phase IV randomized trial
Source: Medicine (Baltimore). 2023 Mar 31;102(13):e33367. doi: 10.1097/MD.0000000000033367 (PMC10063282; doi:10.1097/MD.0000000000033367)
Supplement: Supplementary file 2 [file medi-102-e33367-s002.pdf]

Supplemental Table 2: Analysis populations

	BZN SPRAY	BZN LOZENGES
**Safety population**	**181**	**182**
**m-ITT population**	**181**	**182**
a. patients included in the Safety population	181	182
b. minus 8 major protocol violators	7	1
**PP population**	**174**	**181**
a. patients included in the PP population	174	181
b. minus 66 protocol deviatiors	22	44
**m-PP population for exploratory purposes after 120 minutes**	**152**	**137**
