# Supplementary material for: Comparative evaluation of rapidity of action of benzydamine hydrochloride 0.3% oromucosal spray and benzydamine hydrochloride 3 mg lozenges in patients with acute sore throat: A phase IV randomized trial
Source: Medicine (Baltimore). 2023 Mar 31;102(13):e33367. doi: 10.1097/MD.0000000000033367 (PMC10063282; doi:10.1097/MD.0000000000033367)

Supplemental Figure 1: Mean TOTPAR for STRRS from 2 to 240 after administration of benzydamine HCl spray or benzydamine HCl lozenges in m-ITT, PP and m-PP populations.

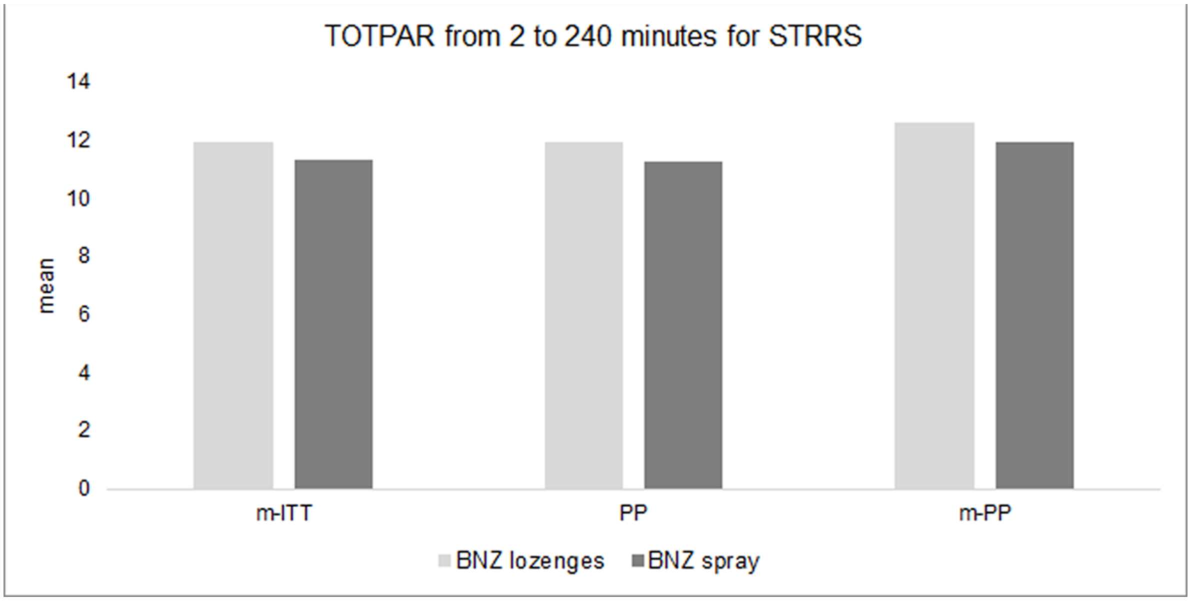

Supplement: Supplementary file 3 [file medi-102-e33367-s003.pdf]
